# Genome-Wide Characterization and Expression Profiling of Putative m^6^A Methylation Regulatory Proteins (Writers and Erasers) in *Ginkgo biloba*

**DOI:** 10.3390/biology15120897

**Published:** 2026-06-08

**Authors:** Yuke Ma, Wenhui Guo, Han Wang, Jingjing Zhang, Meixiang Wei, Chaoyue Shi, Kongshu Ji, Qiong Yu

**Affiliations:** 1 State Key Laboratory of Tree Genetics and Breeding, Nanjing Forestry University, Nanjing 210037, China; mayuke2001@163.com (Y.M.); gwenhui1106@163.com (W.G.); hanwang120902@163.com (H.W.); jjzhang@njfu.edu.cn (J.Z.); weimeixiang@njfu.edu.cn (M.W.); yinwu1999@outlook.com (C.S.); ksji@njfu.edu.cn (K.J.); 2 Co-Innovation Center for Sustainable Forestry in Southern China, Nanjing Forestry University, Nanjing 210037, China; 3 Beijing National Laboratory for Molecular Sciences, Beijing 100190, China

**Keywords:** *N*^6^-methyladenosine, RNA epigenetics, Methyltransferases, Demethylases, *Ginkgo biloba*, abiotic stress

## Abstract

m^6^A is an important RNA modification involved in post-transcriptional regulation in plants. However, putative m^6^A writers and erasers in *G. biloba* remain poorly characterized. In this study, a total of 17 candidate m^6^A regulatory genes, including 8 writers and 9 erasers, were identified through genome-wide analysis. Phylogenetic and structural analyses indicated that these proteins are generally conserved, with some members showing potential functional divergence. Promoter analysis revealed abundant hormone- and stress-responsive cis-elements, and expression profiling showed tissue-specific patterns and dynamic responses to ABA, MeJA, and NaCl treatments, with erasers exhibiting greater transcriptional responsiveness than writers. In addition, protein interaction network and phase separation predictions suggested potential roles in RNA methylation-related processes. These results provide a foundation for further functional studies of m^6^A regulation in *G. biloba*.

## Simple Summary

*N*^6^-methyladenosine (m^6^A) is a common chemical modification on RNA that acts as a regulatory switch controlling gene expression, thereby influencing plant growth and responses to environmental stress. While this mechanism has been widely studied in flowering plants, it remains largely unexplored in gymnosperms, particularly in the ancient tree *Ginkgo biloba* (*G. biloba*). In this study, we identified 17 putative proteins in ginkgo that are responsible for adding and removing this modification. These proteins show conserved evolutionary relationships with those in other related species. Gene expression analysis revealed that these genes are more active in leaves and respond dynamically to environmental changes, including plant hormone treatments and salt stress, with erasers showing stronger responses. These findings provide the first systematic overview of this regulatory system in ginkgo and offer valuable resources for understanding plant adaptation and for future improvement of its medicinal traits.

## 1. Introduction

Eukaryotic messenger RNA (mRNA) contains m^6^A as its most abundant internal modification, which constitutes an essential layer of post-transcriptional gene regulation [1]. This modification is critically involved in several aspects of RNA metabolism, including mRNA stability, splicing, and translation, thereby modulating plant growth, development, and stress responses [2,3,4]. The dynamic and reversible regulation of m^6^A modification is achieved through three categories of proteins: writers, erasers, and readers. Writers, mainly composed of methyltransferase-like (METTL) proteins and their accessory components, catalyze the deposition of m^6^A marks on RNA transcripts [5]. In contrast, erasers, primarily members of the AlkB homolog (ALKBH) family, remove m^6^A modifications and ensure the reversibility of this epigenetic process [6]. Readers, typically represented by YTH domain-containing proteins, specifically recognize m^6^A-modified RNAs and trigger downstream regulatory cascades [7]. Among these regulators, writers and erasers constitute the core machinery responsible for the dynamic regulation of m^6^A modification, whereas readers mainly function as downstream effectors.

The identification and functional characterization of m^6^A regulatory factors have been performed in a wide range of plant species, demonstrating both conserved and species-divergent roles. Prior evidence indicates that these genes are expressed in a tissue-dependent manner and are associated with various abiotic stress responses, including abscisic acid (ABA), salt, and methyl jasmonate (MeJA) [8,9,10,11,12]. In the model plant *Arabidopsis thaliana* (*A. thaliana*) and other plant species, m^6^A writers and erasers play essential roles in plant development and environmental adaptation [3,12]. Recent studies have further expanded the characterization of m^6^A regulatory machinery in woody plants and gymnosperms. In poplar (*Populus* spp.), characterization of m^6^A dynamics under salt stress has demonstrated the stress-responsive functions of RNA methylation regulators in woody plants [11]. In conifer species such as *Larix* spp., the m^6^A methyltransferase complex LkMTA2/LkMTB8 was shown to regulate xylem cell expansion [13], providing functional evidence of m^6^A modification in gymnosperm wood formation. These findings highlight the evolutionary conservation and functional diversification of m^6^A regulatory systems across seed plants. Beyond these functions, emerging evidence suggests that m^6^A regulatory proteins may participate in more complex molecular processes, such as protein–protein interaction (PPI) networks and liquid–liquid phase separation (LLPS), which are increasingly recognized as important mechanisms for organizing RNA metabolism and post-transcriptional regulation [14,15,16,17]. However, despite these advances, regulatory mechanisms involving protein interactions and phase separation remain insufficiently characterized, particularly in gymnosperms.

*G. biloba* is a relic gymnosperm with important evolutionary and medicinal value. Although putative m^6^A reader proteins have been previously identified in this species, providing initial insights into the recognition of m^6^A-modified RNA, a systematic analysis of putative m^6^A writers and erasers is still lacking [18]. Given that writers and erasers directly control the deposition and removal of m^6^A marks, their characterization is important for understanding the dynamic regulation of m^6^A modification. In addition, the potential involvement of putative m^6^A regulatory proteins in PPI networks and phase separation in ginkgo remains to be further explored.

In this study, we performed a genome-wide identification and systematic analysis of putative m^6^A writers and erasers in *G. biloba*. The phylogenetic relationships, gene structures, conserved motifs, chromosomal distribution, and cis-acting regulatory elements were analyzed. Furthermore, expression patterns across different tissues and under various treatments were examined to assess their potential biological roles. In addition, protein interaction networks and phase separation propensity were evaluated to provide preliminary insights into their possible regulatory mechanisms. This study provides a basis for further investigation of epitranscriptomic regulation in *G. biloba*.

## 2. Materials and Methods

### 2.1. Identification and Initial Characterization of m^6^A Writer and Eraser Proteins in G. biloba

Identification of putative m^6^A writer and eraser proteins in *G. biloba* was carried out using two complementary strategies. HMMER (version 3.3.2) was first employed to screen the ginkgo proteome, utilizing conserved domain profiles (MT-A70, WTAP, VIRILIZER, and ALKBH) retrieved from the Pfam database (https://pfam.xfam.org, accessed on 18 August 2025) with an E-value threshold of <1 × 10^−3^ [19,20]. Meanwhile, BLASTP searches (E-value < 1 × 10^−5^) were performed against the ginkgo protein database (accessed on 18 August 2025) using known m^6^A regulatory sequences from *A. thaliana* as queries [21,22]. All resulting candidates were subsequently verified via the Conserved Domain Database (CDD) (https://www.ncbi.nlm.nih.gov/cdd, accessed on 18 August 2025), and sequences lacking characteristic m^6^A-related domains were discarded [23]. Physicochemical properties including molecular weight (MW), theoretical isoelectric point (pI), and GRAVY were calculated using ProtParam (https://web.expasy.org/protparam, accessed on 19 August 2025) [24], while subcellular localization was predicted via WoLF PSORT (https://wolfpsort.hgc.jp, accessed on 19 August 2025) [25].

### 2.2. Genomic Characterization and Comparative Analysis

Chromosomal localization of putative m^6^A-related genes in *G. biloba* was performed using the genome annotation file (GFF format). For each gene ID, corresponding coordinates were extracted and then visualized with TBtools (version 2.320) [26]. Promoter regions spanning 2 kb upstream of the translation start site were retrieved and submitted to the PlantCARE database (http://bioinformatics.psb.ugent.be/webtools/plantcare/html/, accessed on 24 August 2025) for cis-acting element prediction [27]. To explore the evolutionary dynamics of putative m^6^A regulatory genes, we carried out gene duplication and collinearity analyses. Syntenic relationships between *G. biloba* and *Pinus tabuliformis* (*P. tabuliformis*) were detected using MCScanX under default parameters, and the resulting collinear gene pairs were visualized using TBtools (version 2.320) [26].

### 2.3. Phylogenetic Analysis and Structural Characterization

To explore the evolutionary relationships of m^6^A regulatory proteins across major plant lineages, we collected protein sequences from representative species, including *Physcomitrella patens* (*P. patens*), *Selaginella moellendorffii* (*S. moellendorffii*), *P. tabuliformis*, *G*. *biloba*, *A*. *thaliana*, *Vitis vinifera* (*V. vinifera*), *Solanum lycopersicum* (*S. lycopersicum*), *Oryza sativa (O. sativa*), and *Zea mays* (*Z. mays*), covering bryophytes, lycophytes, gymnosperms, eudicots, and monocots (Table S1). Multiple sequence alignment was performed using MAFFT v7 with default parameters [28]. Phylogenetic trees were constructed using IQ-TREE3 [29]. The best-fit substitution model was automatically selected by ModelFinder based on the Bayesian Information Criterion (BIC) [30]. For the putative writer proteins, the JTT+F+R4 model was selected, whereas the VT+F+R5 model was selected for the putative eraser proteins. Branch support was assessed using 1000 ultrafast bootstrap replicates (UFBoot2) [31]. The resulting tree was visualized and annotated using iTOL (https://itol.embl.de, accessed on 25 May 2026) [32].

Protein domain architecture was analyzed using the NCBI CDD (accessed on 20 August 2025) with an E-value threshold of <1 × 10^−2^ [23]. Conserved motifs were identified using the MEME suite (version 5.5.0), with the maximum number of motifs set to 6 and other parameters kept at default settings [33].

### 2.4. Prediction of Protein–Protein Interaction Networks

Potential PPI networks of putative m^6^A regulatory proteins in *G*. *biloba* were predicted using the STRING database (version 12.0, https://cn.string-db.org/, accessed on 30 August 2025). A high-confidence interaction score threshold (>0.7) was applied to ensure reliability of the predicted associations. Functional annotation information, including Gene Ontology (GO) terms enrichment, was also retrieved from the STRING database (Tables S2–S4) [34]. It should be noted that STRING interactions for non-model species such as *G. biloba* are largely inferred via orthology transfer from model species rather than experimentally validated interactions.

The interaction data were saved as tab-separated files and then loaded into Cytoscape (version 3.10.3) to perform network visualization and analysis [35]. Highly interconnected modules in the network were identified by applying the MCODE plugin under default settings [36].

### 2.5. Computational Prediction of LLPS-Associated Properties

To comprehensively evaluate the LLPS-associated properties of putative m^6^A regulatory proteins, four complementary computational predictors were employed. Prion-like domains (PrLDs) were first predicted using the PLAAC platform (http://plaac.wi.mit.edu, accessed on 26 May 2026) with default parameters, which identifies regions with prion-like amino acid composition based on a hidden Markov model [37]. Proteins with a COREscore ≥ 15 were considered high-confidence PrLD candidates. Intrinsically disordered regions (IDRs) were predicted using IUPred2A (https://iupred2a.elte.hu, accessed on 26 May 2026) with the long disorder option [38]. Residues with an IUPred score > 0.5 were considered disordered, and proteins containing at least 50 consecutive disordered residues were classified as having long IDRs. The propensity for spontaneous phase separation was evaluated using FuzDrop (https://fuzdrop.bio.unipd.it, accessed on 26 May 2026) [39]. Proteins with a pLLPS value ≥ 0.6 were considered to possess relatively high LLPS-associated propensity. To further support these predictions, catGRANULE 2.0 ROBOT, a machine learning-based classifier integrating physicochemical properties and AlphaFold2-derived structural features, was employed to assess LLPS-associated features [40]. Proteins with a predicted score > 0.5 were considered putative LLPS-associated proteins. Proteins consistently predicted as positive by at least three of the four methods were considered candidates with relatively high LLPS-associated propensity.

### 2.6. Plant Materials, Growth Conditions, and Stress Treatments

Six-month-old *G. biloba* plants were cultivated under controlled environmental conditions at Nanjing Forestry University. Root, stem, and leaf samples were harvested for expression profiling. For abiotic stress induction, seedlings were supplied with ABA 100 μM, MeJA 100 μM, and NaCl 100 mM [41,42]. Leaf specimens were harvested at 0, 6, 12, 24, and 48 h post-treatment, immediately cryo-preserved in liquid nitrogen, and maintained at −80 °C. All treatments were performed in triplicate, with untreated seedlings serving as the control group.

### 2.7. RNA Extraction and Expression Analysis

Total RNA was isolated from *G. biloba* leaves using a commercially available RNA extraction kit. RNA integrity and concentration were evaluated by agarose gel electrophoresis and a NanoDrop 2000 spectrophotometer (Thermo Fisher Scientific, Waltham, MA, USA). First-strand cDNA was reverse-transcribed from 1 μg of total RNA and served as the template for quantitative real-time PCR (qRT-PCR). Each reaction was carried out with SYBR Green Master Mix under standard thermal cycling conditions and melt curve analysis was subsequently performed to verify amplification specificity. Three biological and three technical replicates were included for all experiments. Transcript levels were normalized to the reference gene *GAPDH*, and relative expression was determined via the 2^−ΔΔCT^ method [43,44,45]. The primer sequences and amplification efficiencies are listed in Table S5 raw Ct values are provided in Table S6, and the stability of the reference gene *GAPDH* is shown in Table S7.

### 2.8. Statistical Analysis

Three independent biological replicates were used for all experiments. Results are expressed as mean ± standard error (SE). Statistical evaluation was carried out using SPSS software (version 27.0, IBM Corp., Armonk, NY, USA), while graphs were produced with GraphPad Prism (version 9.5.0, GraphPad Software, San Diego, CA, USA). Group comparisons were assessed by one-way ANOVA, followed by least significant difference (LSD) test. Statistically significant differences among groups (*p* < 0.05) are denoted by different lowercase letters.

## 3. Results

### 3.1. Identification and Characterization of Putative m^6^A Writers and Erasers in G. biloba

A total of 17 putative m^6^A regulatory proteins, including 8 writers and 9 erasers, were identified in *G*. *biloba* (Table 1). These proteins were named based on their homology in *A*. *thaliana*. The identified proteins exhibited considerable variation in sequence length and physicochemical properties. Protein lengths ranged from 144 to 979 amino acids (aa) for writers and from 241 to 845 aa for erasers. Correspondingly, molecular weights varied from 15.91 to 109.74 kDa in writers and from 27.76 to 91.17 kDa in erasers. The theoretical isoelectric point (pI) values ranged from 5.11 to 7.90 for writers and from 5.80 to 9.65 for erasers, indicating a broader pI distribution among eraser proteins. All identified proteins showed negative GRAVY values, suggesting that they are predominantly hydrophilic, which is consistent with their potential roles in RNA metabolism. Subcellular localization prediction revealed that most of these putative m^6^A regulatory proteins are localized in the nucleus, whereas a minority were predicted to be in the cytoplasm, chloroplasts, or mitochondria, pointing to potential functional divergence.

### 3.2. Phylogenetic Analysis of Putative m^6^A Writers and Erasers

**Figure 1:**
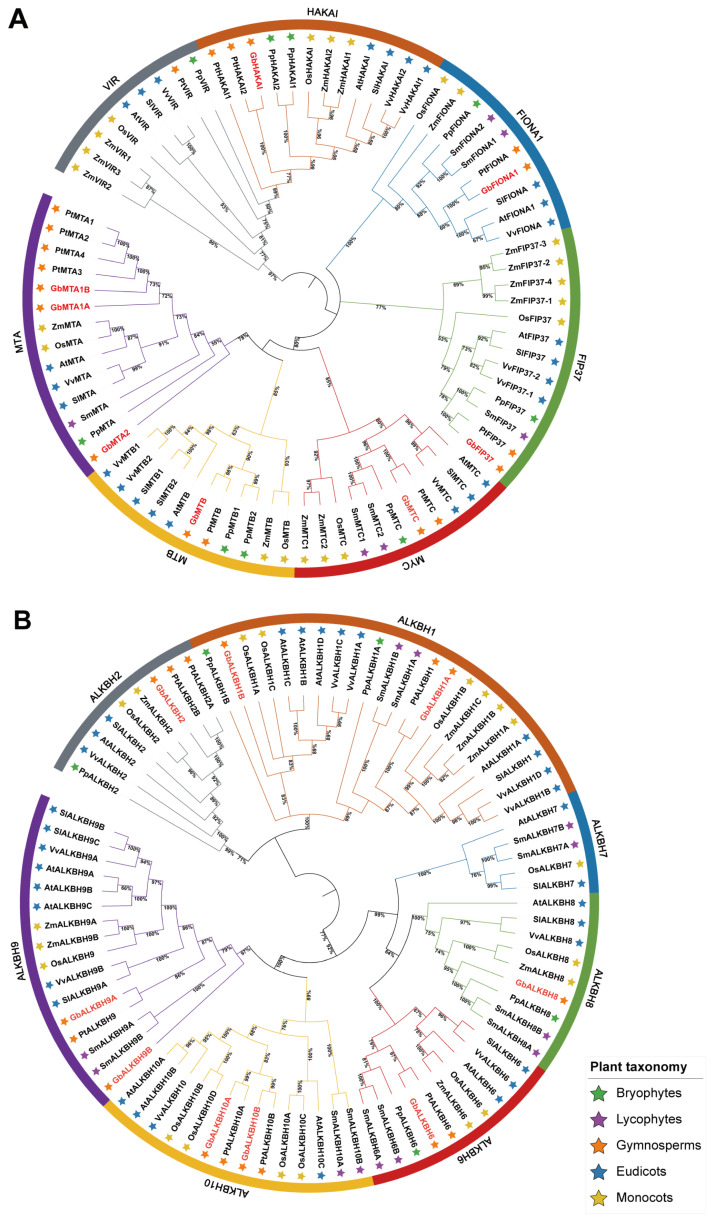
Phylogenetic analysis of putative m^6^A writer and eraser proteins from *G. biloba* and representative plant species. (**A**) Phylogenetic tree of putative m^6^A writer proteins, including members of the MTA, MTB, MTC, FIP37, FIONA1, VIR, and HAKAI subfamilies. (**B**) Phylogenetic tree of putative m^6^A eraser proteins belonging to different ALKBH subfamilies. The ML trees were constructed using IQ-TREE3 with the best-fit substitution model selected by ModelFinder. Branch support was evaluated using 1000 ultrafast bootstrap replicates (UFBoot), and values ≥ 50 are shown on the branches. Different branch and outer-ring colors indicate distinct subfamilies. Stars indicate the plant taxonomic groups represented by each protein. *G. biloba* proteins are highlighted in bold red font.

To investigate the evolutionary relationships of putative m^6^A regulatory proteins, maximum-likelihood (ML) phylogenetic trees were constructed using representative species spanning bryophytes, lycophytes, gymnosperms (including *G. biloba* and *P. tabuliformis*), eudicots, and monocots. The phylogenetic analysis of putative m^6^A writer proteins showed that these proteins could be classified into seven conserved subfamilies, including MTA, MTB, MTC, FIP37, FIONA1, VIR, and HAKAI (Figure 1A). Members within each subfamily generally clustered together with relatively high bootstrap support, indicating that the major components of the m^6^A methyltransferase machinery are highly conserved during land plant evolution. Most *G. biloba* writer proteins clustered preferentially with homologs from the gymnosperm species *P. tabuliformis*, reflecting the evolutionary conservation of these proteins within gymnosperms. Proteins from monocots and eudicots generally formed distinct subclades within each family, whereas bryophyte and lycophyte proteins occupied relatively basal positions, consistent with the established evolutionary relationships among plant lineages. Notably, while a VIR homolog was identified in *P. tabuliformis*, no corresponding homolog was detected in *G. biloba*, which may reflect lineage-specific divergence or incomplete retention in the sampled gymnosperms.

A separate phylogenetic tree was constructed for putative m^6^A eraser proteins (Figure 1B). The ALKBH proteins were grouped into several conserved clades, including ALKBH1, ALKBH2, ALKBH6, ALKBH8, ALKBH9, and ALKBH10, indicating substantial diversification of the ALKBH family during plant evolution. Similar to the writer proteins, most *G. biloba* ALKBH proteins clustered closely with homologs from *P. tabuliformis*, supporting the evolutionary conservation of putative m^6^A eraser proteins in gymnosperms. Several ALKBH clades showed clear separation between monocot and eudicot proteins, whereas bryophyte and lycophyte members were generally located near the basal branches of the phylogenetic tree. Similar to the observation for VIR, no ALKBH7 homolog was recovered from either *G. biloba* or *P. tabuliformis*, suggesting possible lineage-specific divergence within the sampled gymnosperms. Additionally, some ALKBH subfamilies displayed unequal numbers of members among different plant lineages, suggesting possible lineage-specific expansion or functional divergence during the evolution of putative m^6^A eraser proteins.

### 3.3. Conserved Domain and Motif Analysis of Putative m^6^A Writers and Erasers

**Figure 2:**
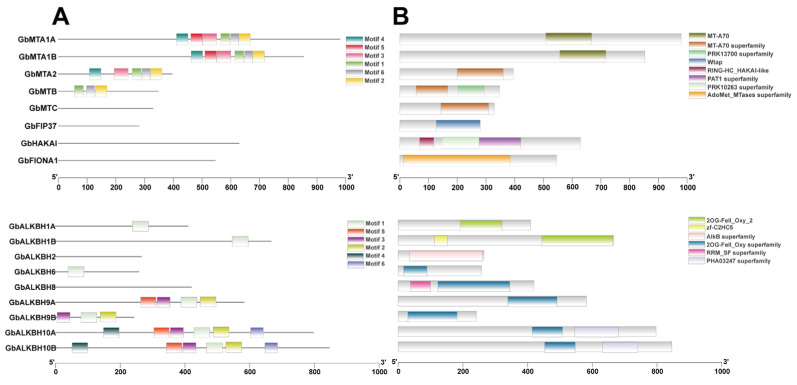
Structural characterization of putative m^6^A writer and eraser proteins in *G*. *biloba*. (**A**) Conserved motif composition of putative m^6^A regulatory proteins identified by MEME. Different colored boxes represent distinct motifs. (**B**) Conserved domain architecture of putative m^6^A regulatory proteins predicted based on CDD analysis. Conserved domains are indicated by specific annotations (e.g., MT-A70, AlkB). Protein names are shown on the left.

To further characterize the structural features of m^6^A regulatory proteins in *G*. *biloba*, conserved motifs and domain architectures were analyzed (Figure 2). Motif analysis revealed distinct distribution patterns among different protein subfamilies (Figure 2A). In the writer group, GbMTA1A and GbMTA1B contained six conserved motifs, whereas GbMTA2A possessed five motifs but lacked motif 5, indicating a relatively high degree of structural conservation among MTA family members. GbMTB contained three conserved motifs, while no common conserved motifs were detected in GbMTC, GbHAKAI, GbFIP37, or GbFIONA1 under the selected MEME parameters. To further verify this result, MEME parameters were optimized by expanding the E-value threshold and reducing the motif width range; however, the results remained unchanged. Considering the principle of MEME analysis, the absence of detectable shared motifs is likely attributable to the high sequence divergence of these proteins relative to the core MTA/MTB proteins, resulting in limited conservation of short peptide regions. Similarly, in the eraser group, GbALKBH9A, GbALKBH10A, and GbALKBH10B contained six conserved motifs, whereas GbALKBH9B possessed only three motifs. In contrast, GbALKBH1A, GbALKBH1B, and GbALKBH6 contained only motif 1, while no conserved motifs were identified in GbALKBH2 or GbALKBH8. Similar to several writer subfamilies, the absence of detectable motifs in GbALKBH2 and GbALKBH8 may reflect substantial sequence divergence and reduced conservation of short motif regions within these proteins.

Domain analysis further confirmed that all identified proteins contained characteristic m^6^A-related functional domains (Figure 2B). Writer proteins in the MTs subfamily harbored the conserved MT-A70 domain, while GbFIP37, GbHAKAI, and GbFIONA1 contained WTAP, RING-HC_HAKAI-like, and AdoMet_MTases domains, respectively. In addition to these canonical domains, some proteins possessed additional domains, such as the PRK13700 superfamily in GbMTB and multiple domains in GbHAKAI, including PRK10263 and PAT1-related domains, suggesting potential functional diversification. All eraser proteins contained the conserved 2OG-Fell_Oxy and AlkB domains. Additionally, several members possessed extra domains, including the PHA03247 domain in GbALKBH10A and GbALKBH10B, the zf-C2HC5 domain in GbALKBH1B, and the RRM domain in GbALKBH8, which is typically associated with RNA binding.

### 3.4. Chromosomal Distribution and Synteny Analysis of m^6^A Writers and Erasers

**Figure 3:**
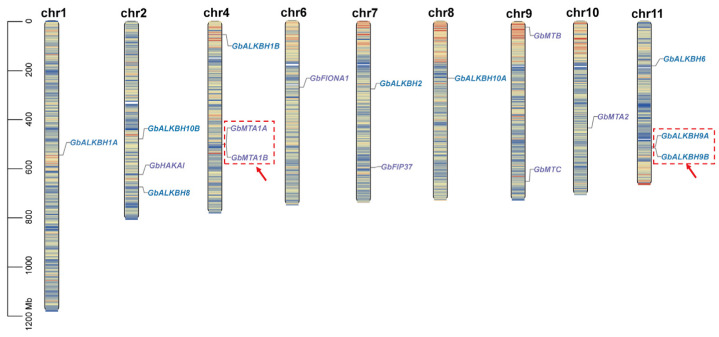
Chromosomal distribution of m^6^A writer and eraser genes in *G*. *biloba*. m^6^A writer genes are indicated in purple, and m^6^A eraser genes are shown in blue. The internal lines within each chromosome represent gene density. Different colors indicate gene density: red corresponds to regions with high gene density, while blue represents low gene density. Chromosome lengths are shown on the left with a scale in megabases (Mb). Tandemly duplicated gene pairs (*GbMTA1A*-*GbMTA1B* on chromosome 4 and *GbALKBH9A*-*GbALKBH9B* on chromosome 11) are highlighted by red dashed boxes and indicated by arrows.

**Figure 4:**
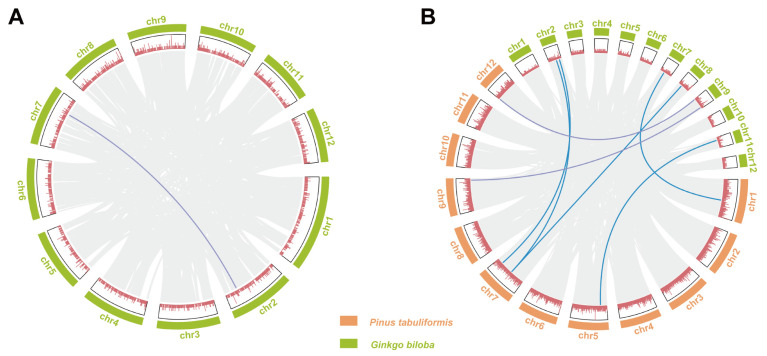
Synteny analysis of m^6^A writer and eraser genes. (**A**) Interspecies collinearity between *G*. *biloba* and *P*. *tabuliformis*. (**B**) Intraspecies collinearity within *G*. *biloba*. Orange and green bars represent chromosomes of *P*. *tabuliformis* and *G*. *biloba*, respectively. Purple lines represent m^6^A writer genes, whereas blue lines represent m^6^A eraser genes. Red lines within the inner circle of chromosomes represent gene density; higher peaks correspond to higher gene density.

The chromosomal distribution of m^6^A writer and eraser genes in *G*. *biloba* was examined (Figure 3). These genes were unevenly distributed across nine chromosomes, with a relatively small number of genes located on each chromosome. Two pairs of tandemly duplicated genes were identified, including *GbMTA1A*–*GbMTA1B* on chromosome 4 and *GbALKBH9A*–*GbALKBH9B* on chromosome 11. Collinearity analysis revealed only one pair of segmentally duplicated genes within the *G*. *biloba* genome (Figure 4A and Table S8). Interspecies collinearity analysis between *G*. *biloba* and *P*. *tabuliformis* identified seven orthologous gene pairs (Figure 4B and Table S8). Among these, two pairs belonged to the writer group (*GbMTB* and *GbMTC*), while five pairs belonged to the eraser group, including *GbALKBH2*, *GbALKBH6*, *GbALKBH8*, *GbALKBH10A*, and *GbALKBH10B*. These results suggested that segmental duplication has played a limited role in the expansion of m^6^A regulatory genes in *G*. *biloba*, whereas interspecies collinearity indicates a relatively conserved evolutionary relationship with other gymnosperms.

### 3.5. Functional Enrichment Analysis of PPI Networks

**Figure 5:**
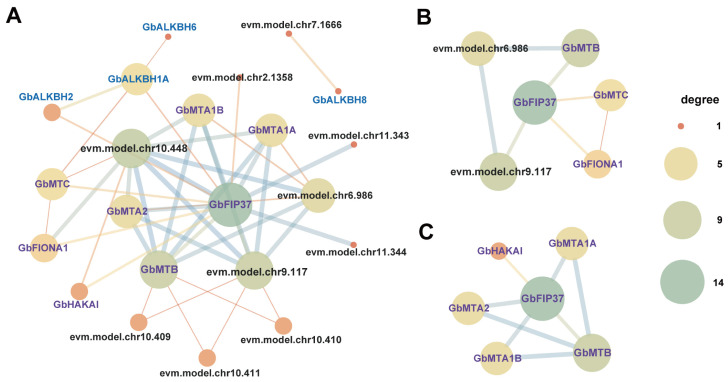
PPI networks of m^6^A writer and eraser proteins in *G*. *biloba*. (**A**) Global PPI network constructed based on STRING analysis (confidence score > 0.7). (**B**) Module 1. (**C**) Module 2. m^6^A writer proteins are shown in purple, m^6^A eraser proteins in blue, and non-m^6^A-associated proteins in black. Node size and color gradient represent degree centrality (range: 1–14), as indicated in the legend. Line color and thickness indicate interaction strength; blue denotes strong interactions, orange weak ones.

The PPI network was constructed based on 17 identified m^6^A regulatory proteins in *G*. *biloba*. A total of 11 m^6^A-related proteins and 9 non-m^6^A-associated proteins were included in the interaction network (Figure 5A). Notably, GbALKBH8 showed an independent interaction with a single protein (evm.model.chr7.1666), which was not integrated into the main network. It should be noted that STRING interactions for non-model species such as *G. biloba* are largely inferred through orthology transfer from model species rather than direct experimental validation. Therefore, the predicted PPI network presented in this study should be regarded as a preliminary computational framework for potential functional associations rather than definitive biological interaction evidence.

GO enrichment analysis of the predicted interaction network revealed enrichment of terms associated with mRNA methylation, RNA methylation, and RNA modification. In the molecular function category, enriched terms included methyltransferase activity, demethylase activity, and RNA binding, whereas cellular component analysis showed enrichment in the RNA *N*^6^-methyladenosine methyltransferase complex and nuclear speck (Table S2).

Further analysis using MCODE identified two major modules within the network (Figure 5B,C). Module 1 was primarily enriched in mRNA methylation–related processes and methyltransferase activity, whereas Module 2 was enriched in both mRNA methylation and developmental processes, including embryo development and post-embryonic development (Tables S2 and S4).

### 3.6. Computational Prediction of LLPS-Associated Features

To evaluate the LLPS-associated properties of putative m^6^A regulatory proteins, four complementary computational predictors were employed, including PLAAC, IUPred2A, FuzDrop, and catGRANULE 2.0 (Table 2). PLAAC identified a high-confidence prion-like domain only in GbHAKAI (COREscore = 19.245) (Figure S1). IUPred2A detected long intrinsically disordered regions (≥50 aa) in 15 of the 17 proteins, with GbALKBH10B showing the longest IDR (~580 aa). FuzDrop predicted relatively high LLPS-associated propensity (pLLPS ≥ 0.6) for ten proteins, with GbALKBH10B, GbHAKAI, and GbALKBH2 showing the highest values (>0.99). catGRANULE 2.0 predicted LLPS-associated features for most proteins (score > 0.5). Integrating the four computational approaches, GbHAKAI was the only protein supported by all four predictors, suggesting relatively high LLPS-associated propensity. Six proteins (GbALKBH10B, GbALKBH2, GbMTA1B, GbALKBH1B, GbALKBH10A, and GbMTB) were supported by three methods, whereas several additional proteins were supported by one or two methods. These results suggest that multiple putative m^6^A regulatory proteins in *G. biloba* possess computationally predicted LLPS-associated features, with GbHAKAI representing the most strongly supported candidate.

### 3.7. Cis-Acting Element Analysis in the Promoter Regions of Putative m^6^A Writers and Erasers

**Figure 6:**
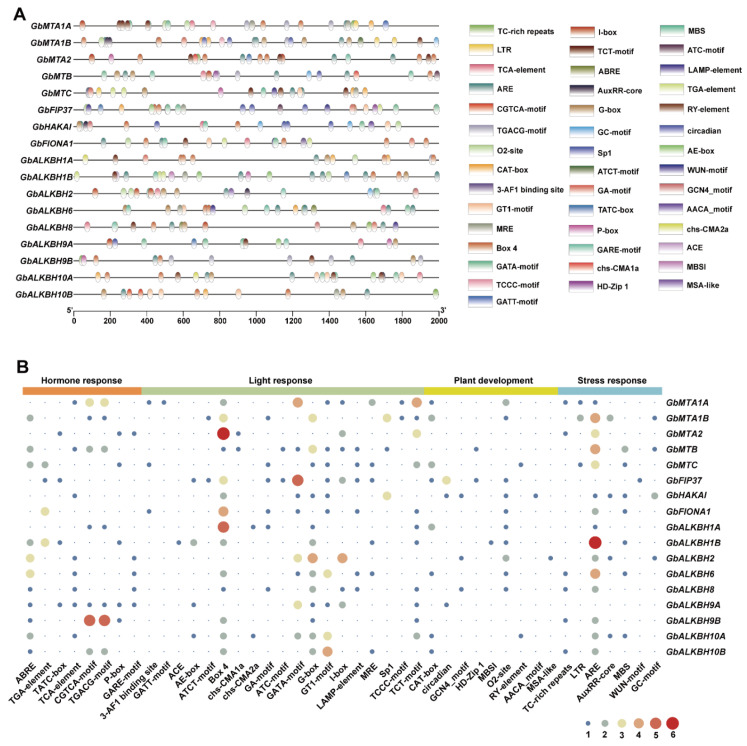
Analysis of cis-acting regulatory elements in the promoter regions of *G. biloba* putative m^6^A writer and eraser genes. (**A**) Distribution of 43 predicted cis-acting elements across the 17 identified putative m^6^A regulatory genes. Different colored boxes represent individual regulatory motifs. (**B**) Classification and quantification of cis-acting elements, with circle size and color indicating the number of occurrences in the promoters.

To investigate the transcriptional regulatory networks of putative m^6^A writer and eraser genes in *G. biloba*, we analyzed the 2 kb promoter regions upstream of the 17 identified m^6^A regulatory genes. A diverse array of cis-acting elements was identified, which were categorized into hormone response, light response, plant development and stress response (Figure 6 and Table S9). Hormone-responsive motifs were widely distributed, with each gene containing at least two such elements. ABRE (ABA-responsive) and CGTCA/TGACG-motifs (MeJA-responsive) were the most prevalent, present in 64.71% and 41.18% of the promoters, respectively. Notably, *GbALKBH9B* harbored a cluster of 10 MeJA-related elements, suggesting a prominent role in jasmonate-mediated signaling. Beyond hormonal control, the promoters were significantly enriched with stress-inducible sequences, including the anaerobic induction element ARE (found in 88.24% of genes) and the drought-responsive MBS (found in 41.18% of genes), which was particularly abundant in *GbALKBH1B* and *GbMTB*. Furthermore, light-responsive motifs, including Box 4 and G-box, were present in all analyzed promoters, with the highest enrichment observed in GbMTA2 and GbALKBH1A. Additionally, development-related motifs such as CAT-box were widespread, indicating that these putative m^6^A regulatory genes may act as key molecular switches integrating growth programs with environmental adaptation in *G. biloba*.

### 3.8. Tissue-Specific Expression Patterns of Putative m^6^A Writers and Erasers

**Figure 7:**
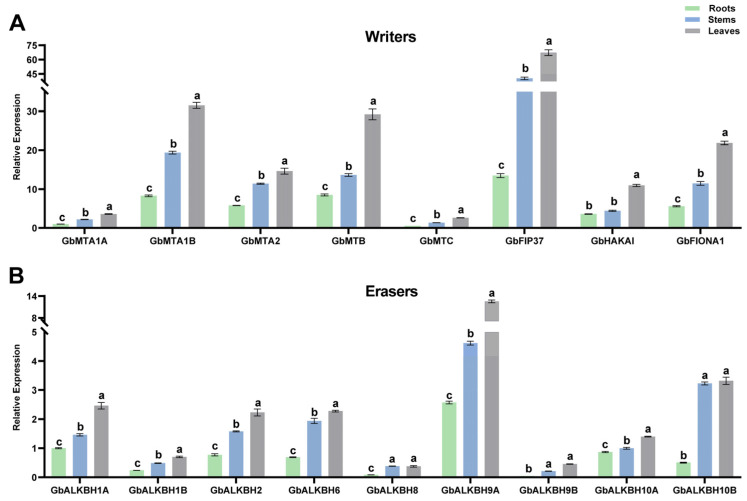
Tissue-specific expression analysis of putative m^6^A regulatory genes in *G*. *biloba*. (**A**) shows writer genes, and (**B**) shows eraser genes. Expression levels of each gene across different tissues (roots, stems, and leaves) were determined by qRT-PCR. For visualization, values were normalized to *GbMTA1A* (roots) for writer genes and *GbALKBH1A* (roots) for eraser genes. Different lowercase letters (a, b, c) indicate significant differences among tissues (*p* < 0.05, one-way ANOVA followed by LSD test), while the same letters denote no significant difference. Data are presented as mean ± SE from three biological replicates.

To investigate the spatial expression patterns of putative m^6^A regulatory genes in *G*. *biloba*, transcript levels in roots, stems, and leaves were analyzed by qRT-PCR (Figure 7). For visualization, expression values were normalized to the level of *GbMTA1A* in roots for writer genes and *GbALKBH1A* in roots for eraser genes. Most genes exhibited a consistent tissue-specific expression pattern, with the highest expression levels in leaves and the lowest in roots, indicating that m^6^A-related processes may play important roles in leaf development and associated physiological processes. One-way ANOVA revealed significant differences among tissues for most genes (*p* < 0.05), with the majority displaying a clear gradient pattern (leaf > stem > root). Notably, a few genes showed distinct expression profiles. *GbHAKAI* exhibited significantly higher expression in leaves compared to both stems and roots, while no significant difference was observed between stems and roots. In addition, *GbALKBH8* and *GbALKBH10B* showed comparable expression levels in stems and leaves, both of which were significantly higher than in roots. Furthermore, *GbFIP37* and *GbALKBH9A* displayed consistently higher transcript abundance across all examined tissues relative to most other m^6^A regulatory genes.

The magnitude of tissue-specific variation differed between writers and erasers. For writer genes, the fold change between leaves and roots ranged from 2.53-fold (*GbMTA2*) to 5.01-fold (*GbFIP37*), whereas for eraser genes, a broader variation was observed, ranging from 1.61-fold (*GbALKBH10A*) to 10.18-fold (*GbALKBH9B*). These results indicated that, although most m^6^A regulatory genes shared a common expression trend, the extent of tissue-specific regulation varied substantially among different gene members.

### 3.9. Expression Profiles Under Abiotic Stress Treatments

**Figure 8:**
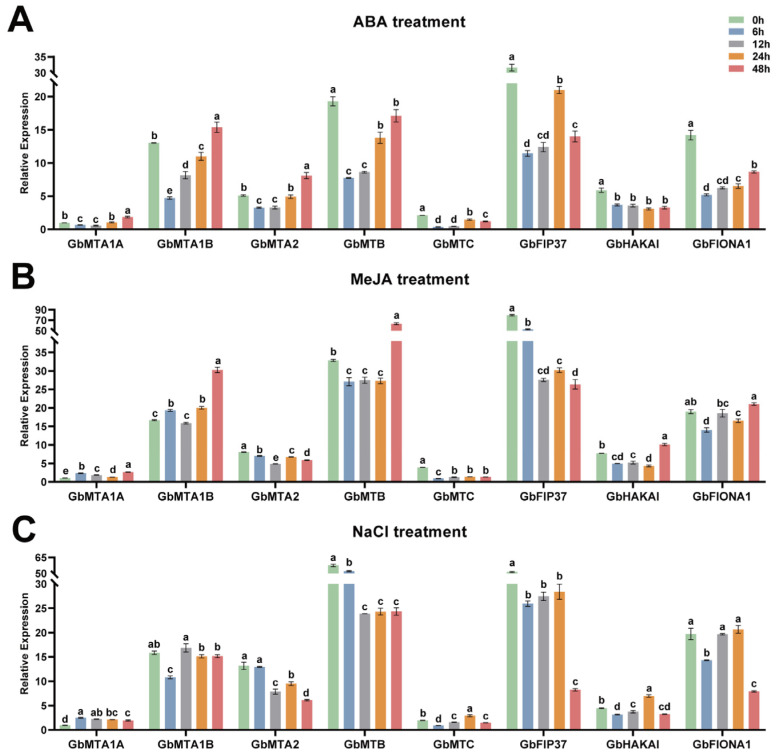
Expression profiles of putative m^6^A writer genes in *G*. *biloba* under different stress conditions. (**A**) ABA treatment; (**B**) MeJA treatment; (**C**) NaCl treatment. In each treatment group, the expression level of *GbMTA1A* at 0 h was set as the reference with a relative value of 1. Data are presented as mean ± standard error (SEM) of three biological replicates. Different lowercase letters indicate significant differences among different time points for each gene under the same treatment (one-way ANOVA, *p* < 0.05).

**Figure 9:**
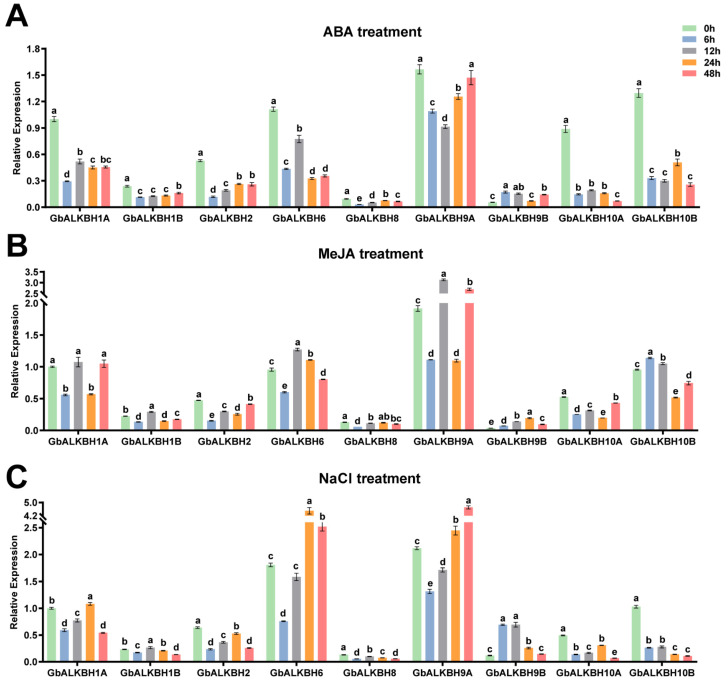
Expression profiles of putative m^6^A eraser genes in *G*. *biloba* under different stress conditions. (**A**) ABA treatment; (**B**) MeJA treatment; (**C**) NaCl treatment. In each treatment group, the expression level of *GbALKBH1A* at 0 h was set as the reference with a relative value of 1. Data are presented as mean ± standard error (SEM) of three biological replicates. Different lowercase letters indicate significant differences among different time points for each gene under the same treatment (one-way ANOVA, *p* < 0.05).

To investigate the responses of m^6^A writer and eraser genes to abiotic stress, their expression patterns under ABA, MeJA, and NaCl treatments were analyzed at five time points (0, 6, 12, 24, and 48 h) using qRT-PCR (Figure 8 and Figure 9). Under ABA treatment, most putative writer genes exhibited a “down–up” expression pattern, with initial suppression at 6 h followed by gradual recovery (Figure 8A). For example, the expression of *GbMTA1A* decreased to approximately 0.45-fold at 12 h and then increased to approximately 2-fold at 48 h. *GbMTA1B*, *GbMTB*, and *GbFIP37* showed sharp declines at 6 h (to 0.3–0.4-fold) before gradually recovering. Putative eraser genes showed a similar but more pronounced suppression at early time points (Figure 9A). Most putative eraser genes, including *GbALKBH1B*, *GbALKBH2*, and *GbALKBH6*, declined sharply at 6 h (to 0.1–0.4-fold) and remained relatively low throughout the time course. Notably, *GbALKBH9A* displayed relatively stable expression, while *GbALKBH9B* showed a transient increase at 6 h (to approximately 3-fold) before declining. These transcriptional responses appear to be largely consistent with the presence of ABRE motifs in the promoter regions (Figure 7), suggesting that ABA-responsive elements may contribute to the observed expression patterns.

Under MeJA treatment, putative writer and eraser genes exhibited diverse expression patterns without a consistent trend (Figure 8B and Figure 9B). Among writer genes, *GbMTB* and *GbFIP37* showed relatively large expression changes, while *GbMTA2*, *GbMTC*, and *GbFIONA1* remained relatively stable (Figure 8B). Among eraser genes, *GbALKBH9A* showed marked upregulation at 12 h, and *GbALKBH9B* exhibited sustained upregulation from 6 h to 24 h (peaking at approximately 5-fold), while most other eraser genes remained at relatively low levels (Figure 9B). These responses appear consistent with the presence of MeJA-responsive elements (CGTCA/TGACG motifs) in the promoter regions of these genes (Figure 7), suggesting that jasmonate signaling may regulate their expression.

Under NaCl treatment, putative eraser genes displayed more pronounced transcriptional changes than putative writer genes (Figure 8C and Figure 9C). While most writer genes exhibited relatively stable or moderately fluctuating expression, several eraser genes showed stronger induction. Notably, *GbALKBH6* showed an increase at 24 h (approximately 2.5-fold), and *GbALKBH9A* exhibited upregulation at 48 h (approximately 2.2-fold). *GbALKBH9B* showed a rapid early response, with a sharp increase at 6 h. Conversely, *GbALKBH10A* and *GbALKBH10B* were consistently downregulated. Taken together, these results suggest that putative m^6^A regulatory genes exhibit stress-specific and temporally dynamic expression patterns, with eraser genes showing greater sensitivity.

## 4. Discussion

m^6^A modification is a key epitranscriptomic mechanism involved in RNA metabolism, development, and stress adaptation [1,2,3,4]. Although extensively characterized in angiosperms, studies in gymnosperms remain limited. Here, we performed a genome-wide analysis of putative m^6^A writer and eraser proteins in *G. biloba*, identifying 17 candidate proteins (8 writers and 9 erasers). The number and classification of these proteins are generally comparable to those reported in other seed plants, suggesting evolutionary conservation of the core m^6^A regulatory machinery across plant lineages [8,9,10,11,12].

Phylogenetic analysis indicated that the putative m^6^A regulatory proteins could be classified into several conserved subfamilies, including MTA, MTB, MTC, FIP37, FIONA1, HAKAI, and multiple ALKBH clades (Figure 1). Most *G. biloba* proteins clustered closely with homologs from *P. tabuliformis*, supporting the evolutionary conservation of these proteins within gymnosperms. Monocot and eudicot proteins generally formed independent subclades, whereas bryophyte and lycophyte proteins occupied relatively basal positions, consistent with established evolutionary relationships among plant lineages. Notably, no VIR homolog was identified in *G. biloba*, although a corresponding homolog was retained in *P. tabuliformis*, suggesting possible lineage-specific divergence during gymnosperm evolution.

Structural analysis revealed that most putative m^6^A regulatory proteins retained conserved functional domains, including the MT-A70 domain in writers and the AlkB/2OG-Fe(II)-Oxy domain in erasers, indicating substantial structural conservation (Figure 2). Motif analysis showed that members within the same phylogenetic subfamily generally displayed similar motif compositions, further supporting their evolutionary conservation. However, several proteins, including GbMTC, GbFIP37, and GbHAKAI, lacked detectable shared motifs under the selected MEME parameters, which may reflect substantial sequence divergence among different subfamilies.

Interestingly, eraser genes generally showed stronger transcriptional responsiveness to ABA, MeJA, and NaCl treatments than writer genes (Figure 8 and Figure 9). This differential expression pattern may suggest distinct regulatory behaviors of writers and erasers during stress responses. Writers are generally considered to maintain basal m^6^A methylation activity, whereas erasers may contribute to more dynamic regulation of RNA methylation under changing environmental conditions [3,4,5,6,7,8,9,10,11,12]. Similar stress-responsive expression patterns of ALKBH family members have been reported in sorghum and barley under salt stress and pathogen infection [10,12]. In barley, the m^6^A demethylase HvALKBH1B was reported to exhibit phase separation-associated behavior during immune responses [12]. Together, these observations suggest that stress-responsive regulation of putative m^6^A eraser proteins may be evolutionarily conserved across diverse plant lineages.

Cis-acting element analysis revealed that the promoters of putative m^6^A regulatory genes contained numerous stress- and hormone-responsive elements, including ABA-, MeJA-, low temperature-, and drought-related cis-elements (Figure 6). Consistent with these promoter characteristics, expression profiling showed that several genes responded markedly to ABA, NaCl, and MeJA treatments, suggesting potential involvement in environmental stress responses (Figure 8 and Figure 9). Similar stress-responsive expression patterns have also been reported in Arabidopsis, poplar, and other plant species, supporting the conserved role of RNA methylation regulators in stress adaptation.

Tissue-specific expression patterns further indicated possible functional differentiation among putative m^6^A regulatory genes in *G. biloba* (Figure 7). Several writer and eraser genes showed relatively high expression in reproductive tissues, whereas others were preferentially expressed in vegetative organs, similar to observations in other plant species [8,9,10,11,12].

Recent studies have suggested that intrinsically disordered regions and prion-like sequence features are frequently associated with the assembly of biomolecular condensates involved in RNA metabolism [17]. Here, several putative m^6^A regulatory proteins exhibited computationally predicted LLPS-associated features, particularly GbHAKAI and several ALKBH members (Table 2). GbHAKAI was the only protein consistently supported by all four computational predictors, suggesting relatively high LLPS-associated propensity. In addition, protein interaction network analysis suggested that putative m^6^A regulatory proteins may participate in complex RNA regulatory networks associated with RNA processing, development, and stress responses (Figure 5). However, these observations are based solely on computational analyses and therefore require further experimental validation (e.g., enzymatic activity assays, subcellular localization, and biophysical LLPS assays) to confirm their biological significance.

Furthermore, given that *G. biloba* leaves are widely used for the production of medicinal extracts, understanding the epitranscriptomic regulation of stress-responsive genes may provide a foundation for future breeding or cultivation strategies aimed at improving the yield of pharmacologically active compounds under adverse environmental conditions. Collectively, this study expands the current understanding of putative m^6^A regulatory proteins in gymnosperms and provides a valuable foundation for future functional, evolutionary, and epitranscriptomic studies in *G. biloba* and other woody plants.

## 5. Conclusions

It should be noted that the present study is primarily based on genome-wide identification, computational prediction, and expression profiling analyses. The predicted functions of these putative m^6^A regulatory proteins as writers and erasers require further experimental validation, including enzymatic activity assays and m^6^A level measurements. This study presents the genome-wide identification and characterization of putative m^6^A writer and eraser genes in *G. biloba*. A total of 17 candidate genes were identified, and their phylogenetic relationships, structural features, chromosomal distribution, and regulatory elements were systematically analyzed. Expression analyses revealed tissue-specific patterns and dynamic responses to abiotic stresses, with erasers generally showing higher transcriptional responsiveness than writers. These findings suggest that putative m^6^A writers and erasers in *G. biloba* share conserved core features while exhibiting regulatory variation, suggesting potential functional differentiation among gene members. This work provides a useful resource for future studies aimed at elucidating the roles of m^6^A modification in ginkgo development and stress responses. In addition, given that *G. biloba* leaves are rich in medicinal compounds, understanding the regulation of m^6^A modification may inform future epitranscriptome-assisted breeding or cultivation strategies to enhance the production of pharmacologically active ingredients under stress conditions.

## Figures and Tables

**Table 1:** Predicted basic characteristics of putative m^6^A writer and eraser proteins identified in *G. biloba*. The table summarizes gene names, protein length, theoretical isoelectric point, molecular weight (MW), instability index, grand average of hydropathicity (GRAVY) and predicted subcellular localization. Physicochemical properties were calculated using ProtParam, and subcellular localization was predicted using WoLF PSORT.

Type	Gene Name	Gene ID	Protein Length (aa)	Theoretical pI	Molecular Weight (KD)	Instability Index	GRAVY	Subcellular Localization Prediction
Writers	*GbMTA1A*	evm.model.chr4.1641	979	6.61	109.74	42.44	−0.251	Nucleus
	*GbMTA1B*	evm.model.chr4.1642	852	6.55	93.85	40.41	−0.450	Nucleus
	*GbMTA2*	evm.model.chr10.1399	395	7.90	45.04	48.23	−0.541	Mitochondrion
	*GbMTB*	evm.model.chr9.116	347	6.02	37.71	53.16	−0.497	Nucleus
	*GbMTC*	evm.model.chr9.2039	328	6.09	37.67	48.83	−0.338	Cytoplasm
	*GbFIP37*	evm.model.chr7.1789	280	5.11	31.76	56.55	−0.858	Nucleus
	*GbHAKAI*	evm.model.chr7.1648	144	5.49	15.91	63.49	−0.290	Nucleus
	*GbFIONA1*	evm.model.chr6.890	545	5.63	60.91	45.36	−0.368	Nucleus
Erasers	*GbALKBH1A*	evm.model.chr1.1539	408	8.07	45.97	41.85	−0.352	Nucleus
	*GbALKBH1B*	evm.model.chr4.229	665	7.42	74.34	49.65	−0.699	Nucleus
	*GbALKBH2*	evm.model.chr7.897	264	9.65	30.20	36.41	−0.581	Nucleus
	*GbALKBH6*	evm.model.chr11.543	256	6.54	28.50	48.31	−0.225	Nucleus
	*GbALKBH8*	evm.model.chr2.1924	420	6.79	47.14	45.95	−0.275	Nucleus
	*GbALKBH9A*	evm.model.chr11.1267	581	5.80	66.14	49.92	−0.751	Chloroplast
	*GbALKBH9B*	evm.model.chr11.1281	241	8.08	27.76	55.50	−0.180	Cytoplasm
	*GbALKBH10A*	evm.model.chr8.906	796	8.87	86.44	47.32	−0.356	Nucleus
	*GbALKBH10B*	evm.model.chr2.1321	845	8.96	91.17	46.46	−0.541	Nucleus

**Table 2:** Summary of computational predictions of LLPS-associated properties for putative m^6^A writer and eraser proteins identified in *G. biloba*. PLAAC: COREscore ≥ 15 indicates a high-confidence prion-like domain (PrLD); values ≥ 15 are shown in bold. IUPred2A: “Yes” indicates an intrinsically disordered region (IDR) of at least 50 consecutive residues with an IUPred score > 0.5; values in parentheses indicate the length of the longest IDR and the peak disorder score. FuzDrop: pLLPS ≥ 0.6 indicates relatively high LLPS-associated propensity; values ≥ 0.6 are shown in bold. catGRANULE 2.0: scores > 0.5 indicate computationally predicted LLPS-associated features. Supported methods indicate the number of positive predictions among the four computational approaches.

Type	Protein	PLAAC (COREscore)	IUPred2A (Longest IDR ≥ 50 aa)	FuzDrop (pLLPS)	catGRANULE 2.0 (Score)	Supported Methods
Writers	GbMTA1A	NaN	Yes (121 aa, 0.89)	0.519	0.67	2/4
	GbMTA1B	NaN	Yes (241 aa, 0.97)	0.965	0.74	3/4
	GbMTA2	NaN	Yes (50 aa, 0.68)	0.214	0.82	1/4
	GbMTB	NaN	Yes (171 aa, 0.95)	0.950	0.82	3/4
	GbMTC	NaN	Yes (51 aa, 0.60)	0.142	0.41	1/4
	GbFIP37	NaN	Yes (280 aa, 0.92)	0.271	0.70	2/4
	GbHAKAI	19.245	Yes (440 aa, 0.99)	0.997	0.59	4/4
	GbFIONA1	NaN	Yes (115 aa, 0.71)	0.408	0.72	2/4
Erasers	GbALKBH1A	NaN	Yes (60 aa, 0.70)	0.225	0.76	2/4
	GbALKBH1B	NaN	Yes (80 aa, 0.80)	0.972	0.87	3/4
	GbALKBH2	NaN	Yes (111 aa, 0.88)	0.997	0.75	3/4
	GbALKBH6	NaN	Yes (121 aa, 0.94)	0.443	0.15	1/4
	GbALKBH8	NaN	No	0.124	0.67	0/4
	GbALKBH9A	NaN	Yes (500 aa, 0.86)	0.666	0.87	2/4
	GbALKBH9B	NaN	No	0.164	0.34	0/4
	GbALKBH10A	NaN	Yes (340 aa, 0.98)	0.974	0.79	3/4
	GbALKBH10B	NaN	Yes (580 aa, 0.94)	0.998	0.83	3/4

## Data Availability

Data are contained within the article and Supplementary Materials.

## Supplementary Materials

The following supporting information can be downloaded at: https://www.mdpi.com/article/10.3390/biology15120897/s1, Table S1: Detail information of m^6^A regulatory genes used for the construction of phylogenetic trees; Table S2: Main_PPI_Network; Table S3: MCODE_Module_1; Table S4: MCODE_Module_2; Table S5: Primers used for qRT-PCR and their amplification efficiencies; Table S6: Raw Ct values for qPCR analysis of putative m^6^A regulatory genes in *G. biloba*; Table S7: Expression stability of the reference gene *GAPDH* across different tissues and stress treatments in *G. biloba*; Table S8: Homologous gene pairs; Table S9: Detailed information of cis-elements in putative promoter sequences; Figure S1: PLAAC prediction of prion-like domains (PrLDs) in putative m^6^A regulatory proteins in *G. biloba.*

## Abbreviations

The following abbreviations are used in this manuscript:

m^6^A	*N*^6^-methyladenosine
mRNA	messenger RNA
METTL	methyltransferase-like
ALKBH	AlkB homolog
ABA	abscisic acid
MeJA	methyl jasmonate
PPI	protein–protein interaction
LLPS	liquid–liquid phase separation
CDD	Conserved Domain Database
ML	maximum likelihood
GO	Gene Ontology
PrLDs	prion-like domains
qRT-PCR	quantitative real-time PCR
SE	standard error
ANOVA	one-way analysis of variance
LSD	least significant difference
