# Supplementary material for: Genome-Wide Characterization and Expression Profiling of Putative m6A Methylation Regulatory Proteins (Writers and Erasers) in Ginkgo biloba
Source: Biology (Basel). 2026 Jun 8;15(12):897. doi: 10.3390/biology15120897 (PMC13296198; doi:10.3390/biology15120897)
Supplement: Supplementary file 1 [file biology-15-00897-s001.zip › Supplement Figure S1.pdf]

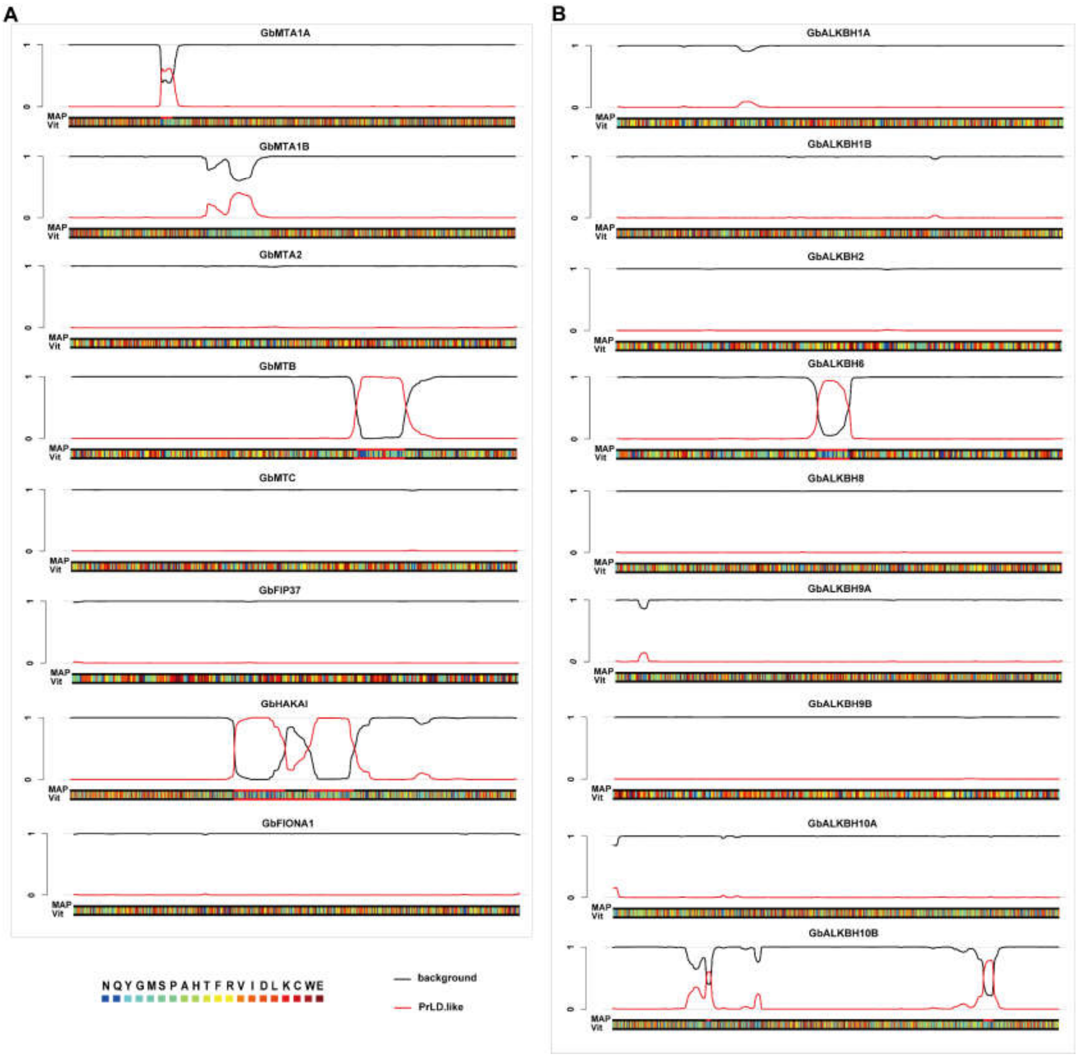

Figure S1. (A) Putative m^{6}A writer proteins. (B) Putative m^{6}A eraser proteins. The red curve represents the PLAAC prion-like domain (PrLD) prediction score, whereas the black curve indicates the background distribution. Regions with elevated PLAAC scores indicate sequence compositions enriched in prion-like characteristics.
